# Identification and detection of a novel human endogenous retrovirus-related gene, and structural characterization of its related elements

**DOI:** 10.1590/S1415-47572009005000082

**Published:** 2009-12-01

**Authors:** Qiaoyi Liang, Jiayi Ding, Shu Zheng

**Affiliations:** Cancer Institute, Second Affiliated Hospital and Ministry of Education Key Laboratory of Cancer Prevention and Intervention, Zhejiang University School of Medicine, HangzhouChina

**Keywords:** endogenous retrovirus, noncoding RNA, RT-PCR, quantitative real-time PCR, transcription

## Abstract

Up-regulation of human endogenous retroviruses (HERVs) is associated with many diseases, including cancer. In this study, an H family HERV (HERV-H)-related gene was identified and characterized. Its spliced transcript lacks protein-coding capacity and may belong to the emerging class of noncoding RNAs (ncRNAs). The 1.3-kb RNA consisting of four exons is transcribed from an Alu element upstream of a 5.0-kb structurally incomplete HERV-H element. RT-PCR and quantitative RT-PCR results indicated that expression of this HERV-related transcript was negatively associated with colon, stomach, and kidney cancers. Its expression was induced upon treatment with DNA methylation and histone deacetylation inhibitors. A BLAT search using long terminal repeats (LTRs) identified 50 other LTR homogenous HERV-H elements. Further analysis of these elements revealed that all are structurally incomplete and only five exert transcriptional activity. The results presented here recommend further investigation into a potentially functional HERV-H-related ncRNA.

Human endogenous retroviruses (HERVs) constitute about 8% of the human genome and are distributed throughout all chromosomes (Lander *et al.*, 2001). H family HERV (HERV-H) is one of the most abundant HERV families in the human genome. It has recently been reported that there are 926 pol-containing HERV-H pro-viruses in the human genome (Jern *et al.*, 2005). The pro-viral structure of HERVs mainly consists of 5' LTR-gag-pro-pol-env-3' LTR, in which ‘LTR' is the long terminal repeat, and the four genes (gag: group-specific antigen, pro: protease, pol: polymerase, and env: envelope) encode structural/functional proteins essential for replication-competent retroviruses. Though most HERV open reading frames (ORFs) have been degraded by deletion or mutation, HERV proteins and transcripts have been detected in reproductive and cancerous cells, such as placenta (Blond *et al.*, 2000), teratocarcinoma cell lines (Lower *et al.*, 1993), breast tumor tissues (Wang-Johanning *et al.*, 2003) and cell lines (Faff *et al.*, 1992), and germ cell tumors (Herbst *et al.*, 1996). In this study, a HERV-H-related gene was identified and characterized. As opposed to what has been described in the literature, this HERV-H-related gene is down-regulated in cancers of the colon, stomach, and kidney.

A 767-bp HERV-related sequence was amplified in kidney samples with primers designed for RT-PCR detection of a gene we had previously been studying (forward: 5'CTCCTGcTCTTTGCTCCGTG3', reverse: 5'GG_TTGTTCTCTGGtGGGCAG3', lowercase for mismatch and ‘_' for lack). A BLAT search at the UCSC Genome Browser revealed that the sequence consisted of three exons with a genomic span of 4.3 kb on chromosome 4p15.2. Sequence analysis of the genomic sequence with the tool RepeatMasker revealed a 5.0-kb HERV-H provirus encompassing the 767-bp sequence. 3' RACE assay revealed this transcript to be polyadenylated at the expected site within the 3' LTR at a CA dinucleotide. 5' RACE results indicated that transcription of this HERV-related transcript did not initiate within the expected 5' LTR, but within an Alu element immediately upstream of the 5' LTR (Figure 1A). Extensive sequence analysis of the 1.3-kb full-length transcript revealed that it lacked protein-coding capacity, thereby inferring that it might belong to the emerging class of noncoding RNAs (ncRNAs). Nucleotide sequences have already been deposited in GenBank with accession numbers EF535612 (767-bp), EF535613 (3' terminus), and EU669866 (the full-length transcript).

The locus of the provirus was Chr4: 23333592-23338589 (hg18) on the reverse strand. Flanked by 5-bp CCCGC direct repeats at both ends and with intact LTRs, the 5.0-kb HERV-H provirus had terminal structures corresponding to integration into the genome through retro-transposition. Pair-wise alignment of the 5.0-kb HERV-H provirus with the 9.0-kb HERV-H consensus element constructed by Jern *et al.* (2005), was carried out with the tool, GeneDoc. The alignment result was then shortened with another tool, Visio, and edited by image editing software. The regions were defined in the same way that Jern *et al.* (2005) had defined the regions of the 9.0-kb HERV-H consensus. Results showed that large fragments of the gag and pol regions, besides nearly the entire env region, were missing in the 5.0-kb HERV-H provirus (Figure 1B). An additional 125-bp segment, lacking in the HERV-H consensus, was found existing in the pre-gag region of 5.0-kb HERV-H. A BLAT search with this 125-bp sequence revealed that similar sequences were contained in many other HERV-H elements, hence suggesting that a HERV-H consensus containing this segment would better represent an ‘original' HERV-H provirus.

RT-PCR and quantitative RT-PCR (qRT-PCR) were carried out in order to analyze the transcription level of the HERV-H-related gene in tissue samples and cancer cell lines. Tumor and adjacent normal tissues of the colon, stomach, liver, lung, and kidney were obtained after surgical resection and stored frozen at -80 °C until RNA extraction (approved by the ethics committee of Zhejiang University and with the formal consent of all the patients involved). Cancer cells were grown in RPMI 1640 supplemented with 10% fetal calf serum. Total RNA was prepared with Trizol reagent (Invitrogen), according to manufacturer's guidelines. RNA samples were always treated with RQ1 RNase-free DNase (Promega) and purified with phenol/chloroform. RNA was then reverse-transcribed into cDNA using M-MLV Reverse Transcriptase (Promega). PCR assays were performed with *Taq* DNA polymerase (Promega) in reaction systems containing 0.2 μM forward and reverse primers each. Thermal cycler parameters were 94 °C 5 min, (94 °C 30 s, 58 °C 30 s, 72 °C 40 s) x 30 cycles for β-actin/36 for target gene, 72 °C 10 min. Sequences of the primers are listed in Table 1. PCR products were separated on a 1.5% agarose gel, purified and directly sequenced.

RT-PCR results indicated that the HERV-H-related transcript was expressed at relatively low levels in kidney tumors when compared to adjacent normal tissues. Detection in colon and stomach samples also indicated that this transcript was expressed at high levels in adjacent normal tissues and at very low levels in tumor tissues (Figure 1C). Transcript levels were further analyzed by TaqMan qRT-PCR, with the glyceraldehyde-3-phosphate dehydrogenase gene (GAPDH) as the endogenous control gene and the average level of colon tumor samples as reference. Sequences of the primers and probes are listed in Table 1. qRT-PCR assays were performed in reaction systems containing 0.3 μM of each primer and 0.2 μM of probe. Thermal cycler parameters of an ABI PRISM 7700 sequence detection system were 50 °C 2 min, 95 °C 10 min, (95 °C 15 s, 60 °C 1 min) x 40 cycles. qRT-PCR assays for detecting both GAPDH and HERV-H4p15.2 were undertaken simultaneously at least three times. Data analysis was carried out according to the ΔΔCt method (Livak and Schmittgen, 2001). Significant expressional differences were detected between tumor and adjacent normal tissues of the three types of cancers by paired-samples *t* tests with the SPSS15.0 tool (Figure 1D). Expression of this transcript was found in neither tumor nor normal tissues of both the liver and lungs (results not shown).

**Figure 1:**
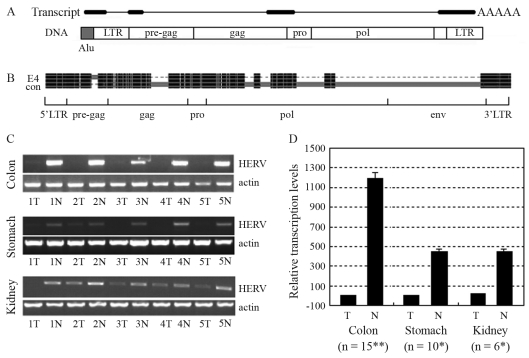
Structure and transcription of the HERV-H-related gene. (A) Schematic for structures of the HERV-related spliced transcript and its template DNA. Thick bars represent exons and thin lines introns. DNA structure of this gene consists of an Alu element (gray) and a HERV provirus (white). Regions of LTRs, pre-gag, gag, pro and pol in the HERV element are labeled. (B) Shortened pair-wise alignment, a result of the 5.0-kb HERV-H element at 4p15.2 (E4) with the HERV-H consensus (con). Black indicates regions of high homogeneity. Gray bars in one accompanied by dash lines in the other indicate existing regions in the first and deletion in the other. Large segments from the gag, pol and env regions are absent in the HERV-H at 4p15.2, whereas an additional 125-bp segment exists in the pre-gag region. (C) RT-PCR detection of the HERV-H4p15-related spliced transcript. The HERV-H-related transcript was transcribed at higher levels in normal than tumor samples of the colon, stomach, and kidneys. (D) Quantitation of the HERV-H4p15-related spliced transcript by qRT-PCR. The HERV-H-related transcript was significantly down-regulated in tumor samples from the colon, stomach, and kidneys. The term ‘n' indicates the number of samples. Expressional differences between tumor and normal samples were verified by *t* tests. **p < 0.001, *p < 0.05. T: tumor; N: normal.

RT-PCR results showed the HERV-H-related gene was not transcribed in colon cancer cells HT29, SW480, SW620 or LS 174T, and only at a low level in RKO (Figure 2A). It was expressed at a low level in the embryonic kidney cell line 293T. No expression was detected in the HepG2 liver cancer cell line, neither in placenta, which has been reported to transcribe HERVs at high levels (Muir *et al.*, 2004). RT-PCR assays produced a smaller band in RKO, SW620, LS 174T and 293T than has ever been seen in tumor or normal tissue samples of the kidneys, colon, stomach, liver or lungs. Sequencing results revealed that this smaller band is another novel HERV-related spliced transcript from 6q24.1 (GenBank accession number EU791617).

Tumor down-regulation of this HERV-H-related gene might be due to the use of an alien promoter rather than the routine 5' LTR. Genes affected by aberrant DNA methylation and chromatin formation include over half of the tumor suppressor genes (Baylin *et al.*, 2001). In order to determine whether the low to no expression of this transcript in cancer is regulated by DNA methylation, cancer cells were treated with the DNA methylation inhibitor 5-aza-2'-deoxycytidine (DAC; Sigma) and the histone deacetylation inhibitor trichostatin A (TSA; Beyotime) as previously described (Cameron *et al.*, 1999): initial treatment with DAC (200 nM) for 48 h, with drug and medium replacement 24 h after beginning the treatment, followed by that of the medium containing TSA (300 nM) for a further 24 h. Cells were also treated with high-dosage DAC (5 μM) or 200 nM DAC only for 72 h, with drug and medium replacement every 24 h.

The target HERV-H-related transcript was expressed at higher levels in HT29 through a combined DAC-and-TSA treatment. Nevertheless, its expression did not increase with either low or high doses of DAC alone, neither was it expressed in SW480 nor SW620 with any type of treatment. Expression of the other HERV-related transcript (smaller band) was induced in HT29 and SW480 with all the treatments. It was induced to a higher level in SW620 by DAC combined with TSA, but was in no way affected by DAC alone (Figure 2B). The results presented herein infer that the HERV-H4p15-related transcript might be regulated by DNA methylation and histone deacetylation. The other HERV-related transcript identified in various cancer cell lines was regulated epigenetically. However, as it was not transcribed in any type of tissue we tested, no further studies were carried out.

BLAT searches with the 5' and 3' LTRs of HERV-H4p15 identified a total of 50 HERV-H elements with lengths ranging from 4690 bp to 6844 bp. Pair-wise alignment was undertaken for each of the 51 elements (including HERV-H4p15) with the HERV-H consensus. According to the segment-deleting patterns, we classified the 51 elements into 10 groups (Supplementary Material, Figure S1). Eight among these contained only one member each. Group V contained three members, one of which was HERV-H4p15. Group III contained the remaining 40 elements, with lengths ranging from 5639 to 6142 bp. The differences in length among members of group III were largely due to part of the pre-gag region. Sequences in this part of the pre-gag region differed greatly among all of the 51 HERV-H elements. As this region is not viral-protein-associated, lack of sequence conservation is not unexpected. So as to simplify the classification of the 51 elements, this variable part of the pre-gag region was not included.

**Figure 2:**
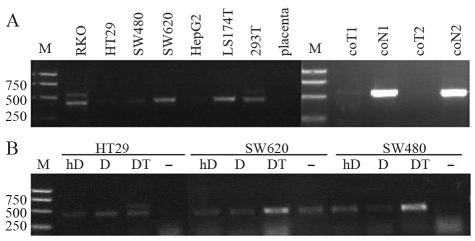
Effects of DNA methylation and histone deacetylation inhibitors on HERV-H4p15 transcription in cancer cells. (A) RT-PCR detection of the HERV-H-related transcript in cancer cells and placenta. The transcript was expressed only in RKO and 293T, and, even then, at low levels (568 bp). A smaller band (435 bp) was detected in RKO, SW620, LS 174T and 293T, which was not seen in colon samples. M, marker; coT, colon tumor; coN, colon normal. (B) RT-PCR detection after treatment with DNA methylation and histone deacetylation inhibitors. The target HERV-H-related transcript was induced in HT29 after combination treatment with DAC and TSA. The smaller band was induced in HT29 and SW480 with all treatments. It was expressed at a higher level in SW620 after combination treatment with DAC and TSA, but was not affected by DAC only. cDNA concentration was normalized by qRT-PCR detection of GAPDH (not shown). M, marker; hD, high-dose DAC (5 μM) for 72 h; D, DAC (200 nM) for 72 h; DT, DAC (200 nM) for 48 h, followed by TSA (300 nM) for a further 24 h; –, no drug control.

Among the over one thousand members of H family HERV, only 18 are relatively complete (Jern *et al.*, 2005). No viral particles produced by HERV-H have ever been found. As shown in Figure S1, the six segments deleted in the most abundant Group III are also correspondingly deleted in some of the other groups, thereby suggesting that segment-deletion in HERV-H elements might be correlated. This common deletion supports the proposal that expansion of H family HERV in the human genome is associated with copying mechanisms (Belshaw *et al.*, 2005). The 340-bp segment deleted in the group-specific antigen region (gag), and commonly corresponding to nucleotide 1933 through 2272 in the HERV-H consensus, belongs to the region encoding the Gag P30 core-shell protein, essential for viral assembly. The protease gene (pro) is not subject to deletion in any of the 51 elements, most likely due to its short extension. The polymerase (pol), which contains reverse transcriptase (RT), RNase H, and integrase (IN) domains, has four sequence segments commonly deleted in many of the 51 elements. The first deleted region in pol corresponds to a 54-aa peptide containing a RT-ZFERV-like conserved domain (a subfamily of RTs and found in sequences similar to those of the intact endogenous retrovirus ZFERV from zebra-fish and Moloney murine leukemia virus). An RNase H conserved domain is contained in the third deleted region in pol, whereas none is found in the second and the fourth belongs to the IN coding region. The envelope protein (env), which consists of the surface unit (SU) and the transmembrane unit (TM), was proved to possess immunosuppressive properties (Mangeney *et al.*, 2001). It is almost entirely deleted in all but the sole member of Group VIII.

Expression of the 51 HERV-H elements was checked. According to results from the UCSC Genome Browser on the Human Mar. 2006 Assembly, 14 of these have spliced expression sequence tags (ESTs). Only five elements produce mRNA sequences, two of which are included in designated genes. Thus, the results demonstrate that most of these structurally incomplete HERV-H elements are inactive.

ncRNA is a global term for transcripts that lack an apparent ORF and do not encode a protein product. Sometimes referred to as mRNA-like ncRNAs, long ncRNAs are transcribed by RNA polymerase II, spliced, polyadenylated and conceivably capped (Erdmann *et al.*, 2000). There are many examples of such long mRNA-like ncRNAs that assume a role during development in both animals and plants (Mattick, 2001). Sequence analysis revealed a lack of any long ORF in the novel HERV-H-related transcript, thereby implying that it might belong to the mRNA-like ncRNAs. Contrary to what has been described in the literature, in this study it was found that the HERV-H-related transcript was down-regulated in cancers of the colon, stomach, and kidney. Furthermore, and interestingly, the expression of this HERV-H-related transcript can be induced upon treatment with DNA methylation and histone deacetylation inhibitors. Considering the abundance of HERV-H elements and their variable expression pattern, a novel proposal is presented that some HERV-H-related RNAs function as regulators to maintain HERV-H expression balance in a cell. Additional studies on this newly identified HERV-H-related non-coding spliced transcript are warranted in order to elucidate its function.

## Supplementary Material

The following online material is available for this article:

Figure S1 Structures of ten types of HERV-H4p15-related HERV-H elements

The material is available as part of the online version of the article from http://wwwscielo.br/gmb.

## Figures and Tables

**Table 1:** Nucleotide sequences of the primers and probes used in this study

Usage/target		Nucleotide sequence (5' to 3')/and matching target site
RT-PCR	HERV-H 4p15.2 (568-bp)	Forward: CCAATTTTAAATCAGGAGCTTGC (exon-exon boundary of exons 2 and 3)
		Reverse: GGTGAGGCAGGGCATATTCA (exon 4)
	
	β-actin (control, 315-bp)	Forward: TCCTGTGGCATCCACGAAACT
		Reverse: GAAGCATTTGCGGTGGACGAT

qRT-PCR	GAPDH (inner control)	Forward: TCGACAGTCAGCCGCATCT
		Reverse: CTTGACGGTGCCATGGAATT
		Probe^i^: FAM-CGTCGCCAGCCGAGCCACAT-TAMRA
	
	HERV-H 4p15.2	Forward: TCCCCTGTCCTCCTGTTCTTT (exon 2)
		Reverse: GAGTGGCTGCCAGGTGAGTT (exon 3)
		Probe: FAM-TGCCCACAGCCCAGGGTTCCT-TAMRA (exon 3)

5'RACE^ii^	cDNA synthesis	5'phosphorylated-CCTGACATTCCTGCC
	
	1^st^ round nested PCR	Forward: GGGTAACTCTCACAGTGGAAGGTT
		Reverse: TGGCTTGGTGGTCAGATTTCT
	
	2^nd^ round nested PCR	Forward: AGCCCTGAGAAACATCGCC
		Reverse: AAGCTCCTGATTTAAAATTGGTGAG

3'RACE^iii^		Forward: CACAGTGGAGGAAGGCAGGAAT

^i^Each probe carried a 5' reporter dye, 6-carboxyfluorescein (FAM) and a 3' quencher dye, 6-carboxytetramethyl-rhodamine (TAMRA). ^ii^5' RACE was performed with the 5' full RACE core set (Takara). ^iii^3' RACE was performed with the 3' RACE System (Invitrogen), other primers also being provided with this kit.
