# Supplementary material for: Identification and detection of a novel human endogenous retrovirus-related gene, and structural characterization of its related elements
Source: Genet Mol Biol. 2009 Dec 1;32(4):704–8. doi: 10.1590/S1415-47572009005000082 (PMC3036903; doi:10.1590/S1415-47572009005000082)
Supplement: Figure S1 — Structures of ten types of HERV-H4p15-related HERV-H elements [file gmb-32-4-704-suppl1.pdf]

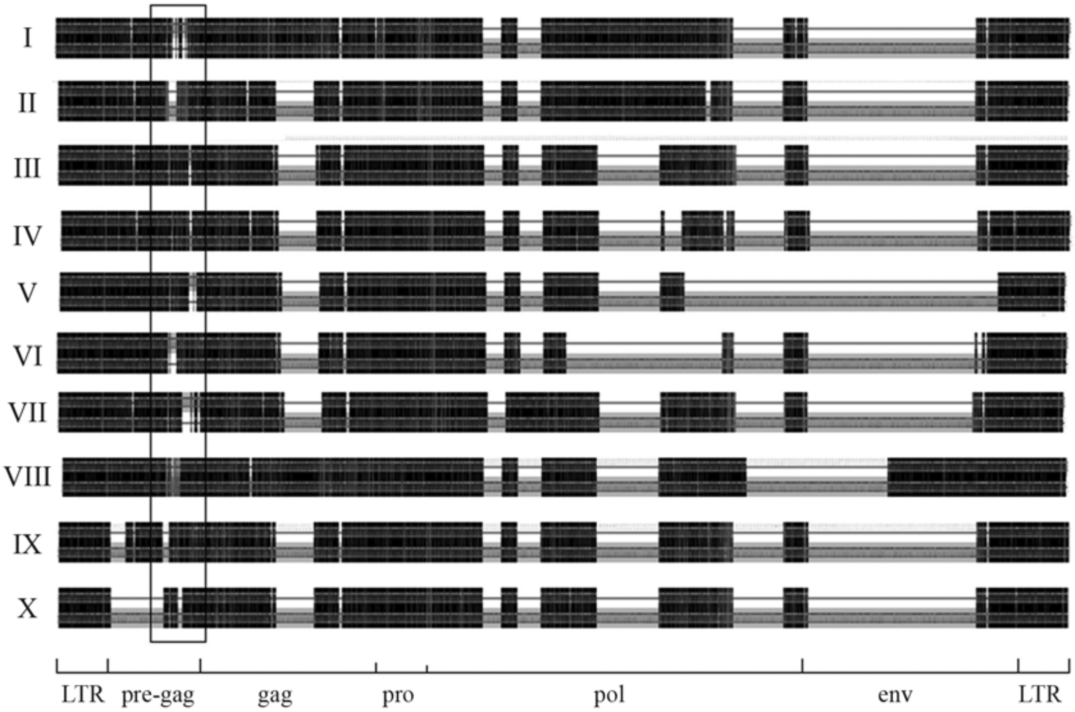

Figure S1 - Structures of ten types of HERV-H4p15-related HERV-H elements. Shown are the pairwise alignment results of the representative HERV-H elements of each group with the HERV-H consensus. Regions of LTRs, pre-gag, gag, pro, pol and env are labeled below. The boxed region is the region in pre-gag which differs greatly among the 51 HERV-H elements.
